# PGC-1α Determines Light Damage Susceptibility of the Murine Retina

**DOI:** 10.1371/journal.pone.0031272

**Published:** 2012-02-13

**Authors:** Anna Egger, Marijana Samardzija, Vithiyanjali Sothilingam, Naoyuki Tanimoto, Christina Lange, Silvia Salatino, Lei Fang, Marina Garcia-Garrido, Susanne Beck, Michal J. Okoniewski, Albert Neutzner, Mathias W. Seeliger, Christian Grimm, Christoph Handschin

**Affiliations:** 1 Biozentrum, Division of Pharmacology/Neurobiology, Biozentrum, University of Basel, Basel, Switzerland; 2 Laboratory for Retinal Cell Biology, Department of Ophthalmology, University of Zurich, Schlieren, Switzerland; 3 Division of Experimental Ophthalmology, Institute for Ophthalmic Research, University of Tübingen, Tübingen, Germany; 4 Department of Biomedicine, University Eye Clinic, University Hospital Basel, Basel, Switzerland; 5 Functional Genomics Center UNI ETH Zurich, Zurich, Switzerland; University of Rochester, United States of America

## Abstract

The peroxisome proliferator-activated receptor γ coactivator 1 (PGC-1) proteins are key regulators of cellular bioenergetics and are accordingly expressed in tissues with a high energetic demand. For example, PGC-1α and PGC-1β control organ function of brown adipose tissue, heart, brain, liver and skeletal muscle. Surprisingly, despite their prominent role in the control of mitochondrial biogenesis and oxidative metabolism, expression and function of the PGC-1 coactivators in the retina, an organ with one of the highest energy demands per tissue weight, are completely unknown. Moreover, the molecular mechanisms that coordinate energy production with repair processes in the damaged retina remain enigmatic. In the present study, we thus investigated the expression and function of the PGC-1 coactivators in the healthy and the damaged retina. We show that PGC-1α and PGC-1β are found at high levels in different structures of the mouse retina, most prominently in the photoreceptors. Furthermore, PGC-1α knockout mice suffer from a striking deterioration in retinal morphology and function upon detrimental light exposure. Gene expression studies revealed dysregulation of all major pathways involved in retinal damage and apoptosis, repair and renewal in the PGC-1α knockouts. The light-induced increase in apoptosis *in vivo* in the absence of PGC-1α was substantiated *in vitro,* where overexpression of PGC-1α evoked strong anti-apoptotic effects. Finally, we found that retinal levels of PGC-1 expression are reduced in different mouse models for *retinitis pigmentosa.* We demonstrate that PGC-1α is a central coordinator of energy production and, importantly, all of the major processes involved in retinal damage and subsequent repair. Together with the observed dysregulation of PGC-1α and PGC-1β in *retinitis pigmentosa* mouse models, these findings thus imply that PGC-1α might be an attractive target for therapeutic approaches aimed at retinal degeneration diseases.

## Introduction

The vertebrate retina translates information of a photon of light into an electrical signal. It is a complex, 7-layered compartment at the innermost part of the eye and is made up of 8 distinct cell types, five of which are neuronal cells. Photons hit photosensitive cells (photoreceptors), which enable phototransduction by conformational change of the photopigment rhodopsin. Distinct photoreceptors allow for vision in light (cone cells) and dark (rod cells) conditions. Subsequent photopigment recovery occurs in the adjacent retinal pigment epithelium (RPE) [1]. The light information is transmitted from the photoreceptors via interneurons (amacrine cells, horizontal cells, bipolar cells) to ganglion cells, which then relay an electric action potential to the optical nerve and ultimately the visual cortex in the brain.

PGC-1 (peroxisome proliferator-activated receptor γ coactivator-1) designates a family of coactivators that comprises PGC-1α, PGC-1β and PGC-1-related coactivator (PRC). These coactivators dock to specific nuclear receptors and other transcription factors, thereby promoting the transcription of target genes, for example those implicated in mitochondrial biogenesis and oxidative phosphorylation (OXPHOS) [2]–[5]. PGC-1α and PGC-1β are mainly expressed in tissues with a high energetic demand, such as skeletal muscle, liver, pancreas, heart, kidney, brain and brown adipose tissue (BAT) [2]–[5]. The expression of PGC-1α and PGC-1β is further regulated in these organs by developmental stimuli and physiological stressors like cold, fasting and exercise. PGC-1α and PGC-1β accordingly regulate tissue-specific functions such as adaptive thermogenesis in brown adipose tissue, gluconeogenesis in the liver or endurance exercise adaptation in skeletal muscle [2]–[5].

Interestingly, despite the well-established link between PGC-1 expression and cellular energetics, the expression and function of the PGC-1 coactivators in the retina, one of the most energy demanding vertebrate organs [6], has not been studied so far, even though a number of cues warrant the analysis of this coactivator family in this organ. The high demand on ATP for physiological retinal function is assured by both glycolysis and oxidative phosphorylation: Neurotransmission relies on glycolysis, sodium transport employs glycolysis and oxidative phosphorylation, whereas phototransduction is mainly fuelled by oxidative phosphorylation to meet energetic demands [7]. In phototransduction, rods and cones are comparable in their energy expenditure in dark conditions, while cones have greater energy demands in the light [8]. Thus, most of the metabolic processes that are important for retinal function are most probably strongly regulated by the PGC-1 coactivators in analogy to other tissues, in particular substrate uptake, fatty acid oxidation and oxidative phosphorylation.

Inversely, pathological conditions of the retina such as age-related macular degeneration and diabetic retinopathy are associated with excess generation of reactive oxygen species (ROS), inflammation and endoplasmic reticulum stress, which result in apoptosis and tissue degeneration [9], [10]. PGC-1α affects these processes by promoting ROS detoxification [11], ameliorating endoplasmic reticulum stress [12] and modulating tissue as well as systemic inflammation [13], [14]. Finally, photoreceptor outer segment renewal involves activation of PPARγ [15], a functional interaction partner of the PGC-1 coactivators in various tissues [16].

Thus, to investigate the potential role of PGC-1α in the retina, we assessed the expression patterns of PGC-1α and PGC-1β in retinal development, adult retina and mouse models for degenerative retinal diseases. Furthermore, we investigated the consequences of genetic ablation of PGC-1α in the retina in terms of changes in gene expression, morphology and function in the basal, dark condition and upon light-induced retinal stress and damage. Finally, we validated some of our findings in retinal cells *ex vivo* with acutely altered levels of PGC-1α. Our findings indicate a crucial role for PGC-1α in tissue protection, in part by inhibition of apoptosis.

## Results

### PGC-1α and PGC-1β are highly expressed in murine retina

**Figure 1:**
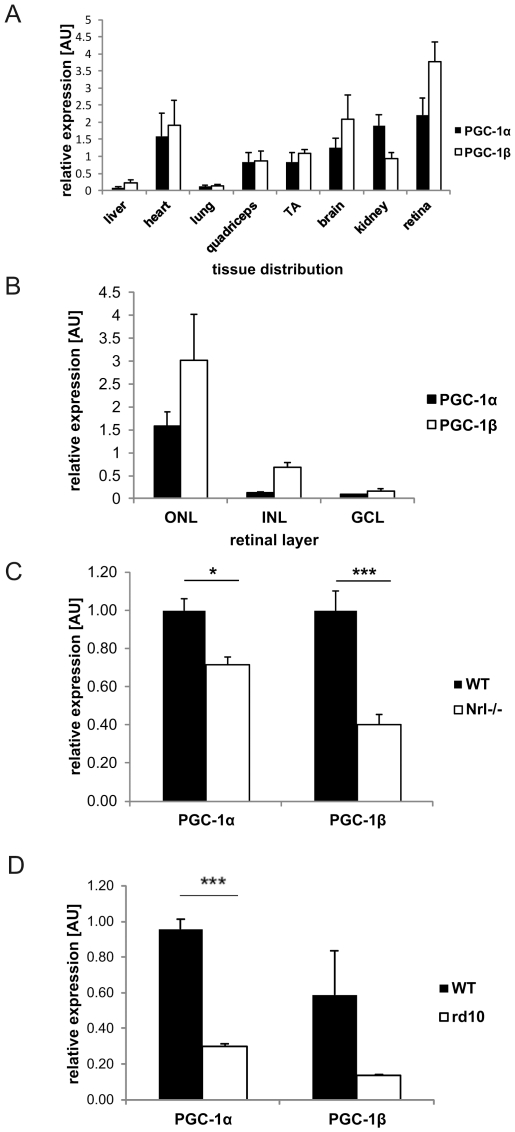
PGC-1α and PGC-1β are strongly expressed in photoreceptors in the retinal outer nuclear layer. Relative mRNA expression levels of PGC-1α and PGC-1β in different mouse tissues evaluated by semi- quantitative real time PCR. β- actin was used as housekeeping gene (HKG). P value was calculated using two tailed Student's T test: * = p<0.05; *** = p<0.001. +SEM **A.** Relative mRNA expression in different tissues from 3 month old wild type male C57BL/6j mice (TA = tibialis anterior). n = 4 **B.** Relative mRNA expression levels of PGC-1α and PGC-1β in different retinal layers from 3 month old wild type male C57BL/6 mice: ONL = outer nuclear layer, INL = inner nuclear layer, GCL = ganglion cell layer. n = 4 **C.** +**D.** Relative mRNA expression levels of PGC-1α and PGC-1β in two retinal disease models: Retina was isolated from *Nrl−*/−, rd10 and age matched control C57BL/6j WT mice. n = 3. **C.** Expression of PGC-1α and PGC-1β in *Nrl−/−* mice at 2 months of age. Data were normalized against C57BL/6j WT mice. **D.** Expression in rd10 mice at 5–6 months of age.

Real-time qPCR-based expression analysis revealed highest PGC-1α and PGC-1β levels in tissues with a great energetic demand. Accordingly, PGC-1α and PGC-1β were found at elevated levels in skeletal and cardiac muscle, brain and kidney. Both coactivators were more moderately expressed in liver and lung. Strikingly, the highest expression levels of all tissues studied were observed in the retina for PGC-1α and PGC-1β (Fig. 1A).

To assess the differential expression pattern of PGC-1α and PGC-1β in distinct retinal compartments, we used laser capture microdissection to separate the different retinal cell layers and subsequently quantified expression via qPCR. PGC-1α and PGC-1β showed their highest expression in the outer nuclear layer (ONL) that harbors rod and cone photoreceptors (Fig. 1B). Although markedly lower compared to ONL, expression of both coactivators was also found in the inner nuclear layer (INL), which contains bipolar, horizontal, amacrine and Müller glia cells, and the ganglion cell layer (GCL), where ganglion and amacrine cells are located (Fig. 1B).

To distinguish between rod and cone-specific expression of the PGC-1 coactivators within the ONL, we determined PGC-1α and PGC-1β levels in neural retinal leucine zipper (*Nrl*) knockout animals, which lack rod photoreceptors but retain cone morphology and function [17] and the rd10 mouse model where rod degeneration is followed by cone degeneration until all photoreceptors are lost by day 60 [18]. The significant reduction of PGC-1α and PGC-1β transcript levels in *Nrl* knockout mice (Fig. 1C) and the rd10 animal model (Fig. 1D) implies that both coactivators are expressed in rods and cones.

### Genetic ablation of Pgc-1α does not compromise morphology and function in the dark adapted, unstressed mouse retina

**Figure 2:**
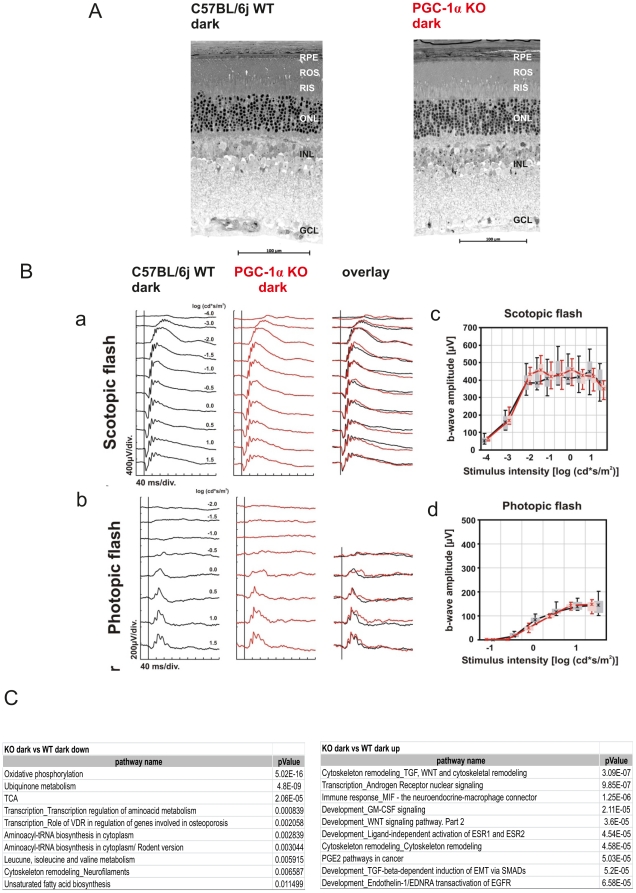
Regular morphology and function in PGC-1α KO mouse retina. Dark adapted, 13 week old PGC-1α KO and C57BL/6j WT were subjected to morphological analysis and electroretinogram (ERG). **A.** Representative retinal morphologic sections from the lower central retina of 13 week old PGC-1α KO and C57BL/6j WT mice: RPE = retinal pigment epithelium, ROS = rod outer segments, RIS = rod inner segments, ONL = outer nuclear layer, INL = inner nuclear layer, GCL = ganglion cell layer. **B.** [**A**] Scotopic (dark-adapted) and [**B**] photopic (light-adapted) single-flash ERGs with increasing light intensities recorded from PGC-1α KO and C57BL/6j WT control mice. The vertical line crossing each trace shows the time point of the light flash. +SEM of n = 4. [**C**] Scotopic and [**D**] photopic b-wave amplitudes from PGC-1α KO and C57BL/6 control mice as a function of the logarithm of the flash intensity. Boxes indicate the 25% and 75% quartile range, whiskers indicate the 5% and 95% quantiles. **C.** Microarray analysis of PGC-1α KO and C57BL/6j WT control mice. Shown are the top 10 pathways up-/downregulated in their mRNA expression of PGC-1α KO and C57BL/6j WT mice, dark exposed. n = 3; * = p<0.05; ** = p<0.01; *** = p<0.001. Statistical significance was calculated using ANOVA test, Benjamin Hochberg corrected. Thresholds for changes in gene expression set at 1.2 (20% above control) or 0.80 (20% below control).

PGC-1α global knockout animals [19] were used to study the function of PGC-1α in the retina. First, we analyzed basal morphology of 13 week- old PGC-1α knockout (KO) and C57 BL/6 WT control mice. Both KO and WT animals revealed an intact, regularly shaped retinal structure (Fig. 2A). To assess basal retinal function, the mice were kept in the dark and then subjected to an electroretinogram (ERG), which tracks the electrical responses of retinal cells to a light stimulus. The resulting a and b and waves reflect the function of the outer and inner layer, respectively. ERG responses were recorded in scotopic (darkness) and photopic (illuminated) conditions to analyze rod and cone function, respectively. In all of these experimental contexts, the PGC-1α KO mice showed regular a and b waves undistinguishable from the WT control animals (Fig. 2B). Finally, in addition to the morphological and functional studies, we investigated whether genetic ablation of the PGC-1α gene in the retina affects global gene expression patterns with gene expression microarrays. Gene ontological analysis revealed primarily a reduction in metabolic pathways in the KO animals, foremost oxidative phosphorylation and citrate cycle (Fig. 2C), similar to results in loss-of-function experiments with PGC-1α in other tissues. Inversely, cytoskeleton remodeling, transforming growth factor beta (*Tgfβ*) and *Wnt* signaling were the most prominent pathways expressed at a higher level in KO compared to WT mice (Fig. 2C).

### Absence of PGC-1α increases light-damage susceptibility

**Figure 3:**
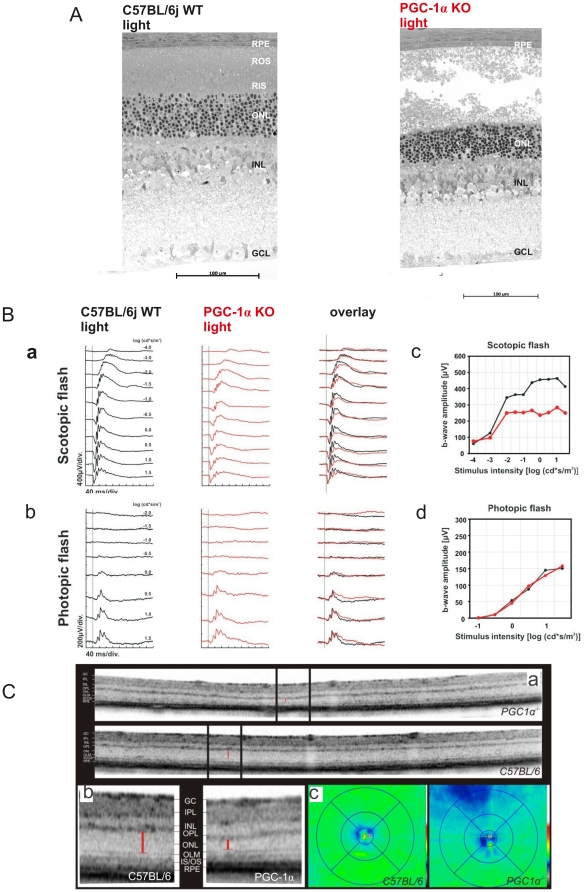
Aberrant morphology and compromised function of PGC-1α KO mouse retina under light stress conditions. PGC-1α *KO* and C57BL/6j WT, aged 13 weeks, were exposed to 15.000 lux of white light for 2 hours and after 24 hours and 10 days of recovery subjected to morphological and functional analysis, respectively. **A.** Representative retinal morphologic sections from the lower central retina of PGC-1α KO and C57BL/6j WT mice. **B.** [**A**] Scotopic (dark-adapted) and [**B**] photopic (light-adapted) single-flash ERGs with increasing light intensities recorded from PGC-1α KO and C57BL/6j WT control mice. The vertical line crossing each trace shows the time point of the light flash. [**C**] Scotopic and [**D**] photopic b-wave amplitudes from PGC-1α KO and C57BL/6 control mice as a function of the logarithm of the flash intensity. (**c**) Optical coherence tomography (SD-OCT): [**A**] comparison of two representative OCT horizontal slices of PGC-1α KO and C57BL/6j WT control mice. [**B**] Magnification of these SD-OCT horizontal slices: left side: C57BL/6, right side: PGC-1α KO; GC = ganglion cell layer, IPL = inner plexiform layer, INL = inner nuclear layer, OPL = outer plexiform layer, ONL = outer nuclear layer, OLM = outer limiting membrane, IS/OS = inner segment/outer segment, RPE = retinal pigment epithelium [**C**] Topography of retinal thickness of C57BL/6 (side) and PGC-1α KO (right) mice calculated from 61 equidistant SD-OCT slices (green = dense; blue = less dense).

While in most organs, the effects of genetic ablation on morphology and function are relatively mild, stress conditions like exercise, cold or fasting greatly exacerbate the phenotype of PGC-1α knockout mouse models [13], [19]. Thus, triggered by our observation of a small effect of PGC-1α knockout on retinal morphology and function in the dark, we sought to study the repercussions of PGC-1α ablation after employing a physiological, retina-specific stressor. For this purpose, PGC-1α KO and WT control mice were exposed to 15.000 lux of white light for 2 hrs, which corresponds to direct sun light exposure. After a 24 hrs recovery period, morphologies of PGC-1α deficient and WT control retinae were analyzed. In stark contrast to the dark-adapted mice (Fig. 2), PGC-1α KO and WT control mice responded markedly differently to a strong light insult. First, most of the rod inner (RIS) and outer (ROS) segments were completely destroyed and most nuclei were pyknotic in the ONL of PGC-1α KO mice (Fig. 3A). Thus, most photoreceptors have lost their light absorption (ROS) and energy-production (RIS) sites. In contrast, the corresponding regions in WT exhibited only occasional pyknotic nuclei (Fig. 3A) and only one out of six wild type animals showed measurable retinal damage, which, however, was still less intense than in PGC-1α KOs (data not shown). In either of the genotypes, the INL and GCL layers were unharmed consistent with previous data showing that light damage causes selective death of photoreceptor cells [20] (Fig. 3A).

Given the nature and extent of retinal degeneration observed in the KO animals, it seemed highly likely that these morphological changes would translate into loss of retinal function. Light- exposed PGC-1α KO mice displayed a blunted scotopic ERG compared to the WT mice with a significant reduction in b wave amplitude 10 days post insult (Fig. 3B). This timeframe was chosen as retina clears injured photoreceptors and returns back to a basal state within 10 days [21]. The photopic response in KO animals remained similar to WT mice, which is consistent with the notion that cones remain unharmed in an acute light- damage paradigm, at least at early timepoints after light damage [22].

Subsequent to the ERG analysis, animals were subjected to spectral domain optical coherence tomography (SD-OCT), which allow for highly sensitive cross sectional analysis of retinal layers, respectively, and thus reveal retinal detachment as well as delicate structural impairments. Cross sectional retinal analysis showed that the KO mice that had a reduced scotopic ERG response also displayed a thinning of the ONL compared to WT controls. (Fig. 3C- abc). This thinning was also observed in the morphological analysis in Fig. 3A. Interestingly however, the morphology indicates an altered arrangement of the nuclei in the ONL to underlie the thinning. Thus, since the total number of nuclei in KO and WT animals is indistinguishable, an increase in relative density is found in the thinner ONL of KO mice (KO: 43 nuclei/mm^2^, WT: 31 nuclei/mm^2^).

### Light-exposed PGC-1α KO animals exhibit reduced expression of phototransduction genes, increased expression of apoptotic and inflammatory markers

**Figure 4:**
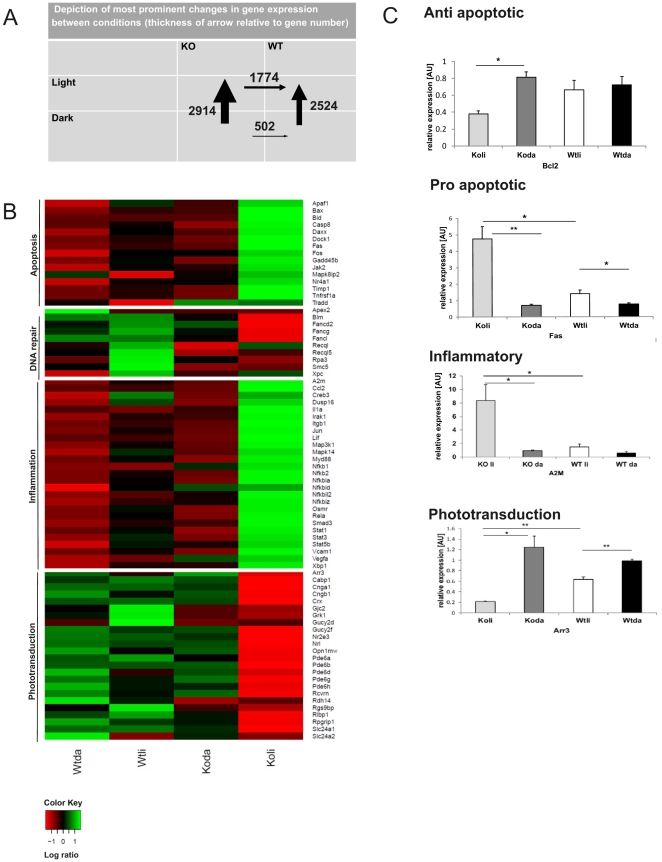
Microarray: Ablation of PGC-1α affects gene expression changes relative to apoptosis, inflammation, protein folding and phototransduction. Microarray analysis of light exposed PGC-1α KO and C57BL/6j WT control mice. +SEM for n = 3 **A.** Number of genes changed significantly between genotypes and conditions: thickness of arrow relates to number of genes changed. **B.** Heat map: expression patterns of a representative selection of genes involved in apoptosis, inflammation, DNA repair and phototransduction. Green = upregulated; red = downregulated; black = unchanged. Data were analyzed with the PARTEK and Bioconductor software suites using Benjamin-Hochberg-corrected ANOVA tests in Panels A and B. Threshold of significant gene expression changes were set at 1.2 fold up- and 0.87 fold down-regulation, respectively. **C.** Semi-quantitative real time PCR validation examples of pro apoptotic, anti apoptotic, inflammatory and phototransduction genes changed significantly in the microarray. 18SrRNA was used as HKG. P value was calculated using two tailed Student's T test: * = p<0.05; ** = p<0.01. +SEM of n = 3; Koli = PGC-1α knockout, light condition, Koda = PGC-1α KO, dark condition, Wtli = C57BL/6 WT, light conditions, Wtda = C57 BL/6 WT, dark condition.

To elucidate the deficits in gene expression in the light-stressed PGC-1α KO animals, we compared gene expression microarrays of WT and KO mice in the control, dark-adapted state and after light exposure, respectively. Interestingly, the influence of the environmental conditioning from dark to light exposure was similar in the two genotypes in regard to the number of regulated genes. However, most of the 2914 genes regulated in the KO animals between dark and light were different from the 2524 genes in the WT animals or were modulated at a significantly higher level. Thus, light- exposed PGC-1α KO and WT mice differed in the expression of 1774 genes, 897 of which were elevated and 877 of which were decreased in KO compared to the WT mice, more than three times the number of genes as observed in the corresponding dark accommodated mouse comparison (Fig. 4A, Fig. S1E). The top regulated genes (Table S1A) and the most affected pathways (Table S1B) that were downregulated in KO mice compared to WT animals still included metabolic processes as observed in the dark condition. Strikingly however, various key processes in retinal function, damage and repair, in particular visual perception, photoreceptor function, DNA damage and repair were now lower in the KO animals compared to controls (Fig. 4B, 4C and Fig. S1A–C). Phototransduction pathway analysis showed that PGC-1α KO mice displayed diminished expression of phototransduction genes in the illuminated state (for example KO light vs WT light: *Nrl, Nr2e3, Arr3, Scn4a, Cnga1*) (Fig. 4B, 4C, Table S1B). Similarly, the expression of many anti-apoptotic genes was reduced in the animals with an ablated PGC-1α gene, including *Fas* (Fig. 4B, 4C and Table S1). Inversely, many inflammatory and pro-apoptotic genes were expressed at a higher level in light-exposed KO mice compared to controls, such as *Bcl2* and *A2M,* respectively (Fig. 4B, 4C). Interestingly, genes involved in extrinsic or intrinsic apoptosis were upregulated in the PGC-1α KO vs WT animals; however, no specific apoptotic pathway was preferentially affected (Table S2A). Finally, only the WT animals exhibited an increase in oxidative phosphorylation gene expression pattern comparing dark adapted to light exposed mice (Table S1B: WTli vs WTda up; KOli vs KOda up).

### PGC-1α expression alleviates apoptosis in retinal cells

**Figure 5:**
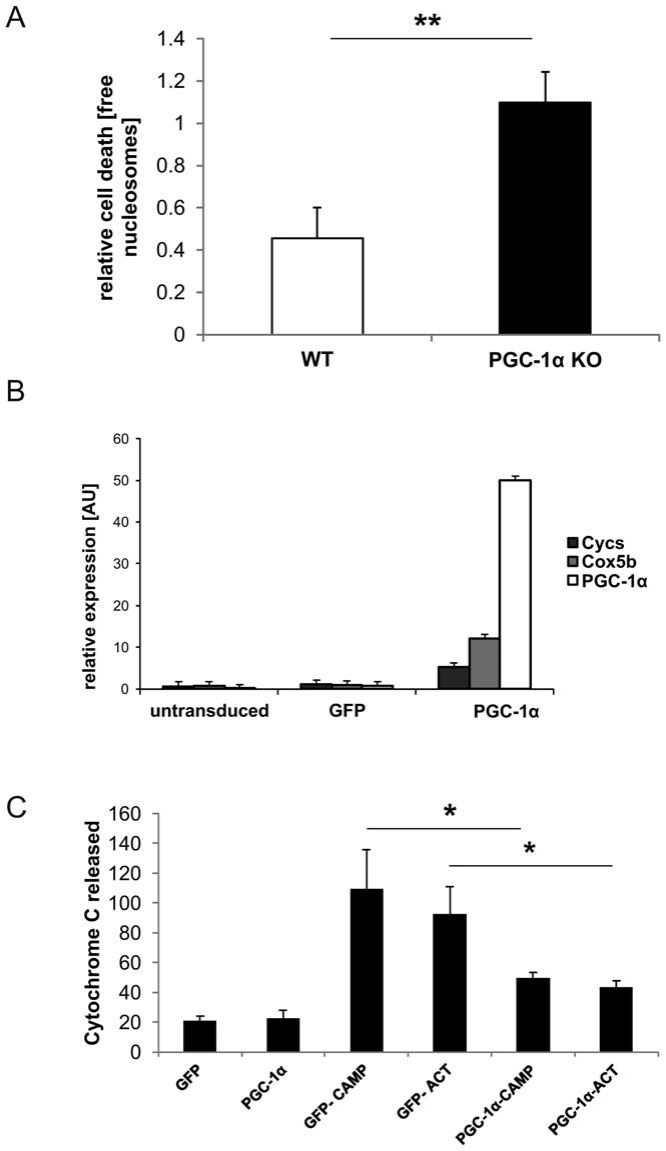
PGC-1α alleviates retinal apoptotic response upon light stress in vivo and in vitro. Relative quantification of cell death. P value was calculated using two tailed Student's T test: * = p<0.05; ** = p<0.01; *** = p<0.001 **A.** PGC-1α KO and C57BL/6j WT mice were exposed to 15'000 lux of white light for 2 hrs. After 24 hrs, retina was extracted and analyzed for free nucleosomes as a measure of cell death via ELISA. +SEM of n = 11 (WT) and n = 15 (PGC-1α KO). **B.** Retinal pigment epithelial cells ARPE-19 were transduced with adenovirus carrying GFP or mouse PGC-1α. Semi-quantitative real time PCR of PGC-1α and its targets *Cox5b* (cytochrome *c* oxidase subunit Vb) and *Cycs* (cytochrome *c*) in PGC-1α versus GFP transduced ARPE-19 cells. 18SrRNA was used as HKG. +SEM of n = 6 **C.** ARPE-19 cells were transduced with adenovirus carrying GFP or PGC-1α, treated with apoptosis inducers actinomycin D or camptothecin and then analyzed for cell death via immunostaining of released cytochrome *c*. ACT = Actinomycin D, CAMP = Campthothecin. 18SrRNA was used as HKG. +SEM of n = 6.

Light exposure of the retina leads to damage and induction of apoptotic pathways. Based on our observation in light exposed animals, we tested whether PGC-1α function is directly involved in the regulation of early events in photoreceptor apoptosis and we therefore quantified cell death in PGC-1α KO and WT control animals following light exposure. In this experimental context, the generation of free nucleosomes was detected in both genotypes, but at significantly higher levels in the PGC-1α KO than in the WT control animals (Fig. 5A). Thus, the deterioration of the KO retina upon light exposure involves an increase in apoptotic events.

To circumvent the potentially confounding effects of a global, life-long absence of PGC-1α in the KO mice that may be compensated *in vivo*, we investigated the effect of acutely modulated PGC-1α in ARPE-19 cells, a retinal pigment epithelial cell line. In these cells, we studied cytochrome *c* release as an early event in intrinsic, mitochondria-related apoptosis. ARPE-19 cells were transduced with adenovirus encoding GFP as control or PGC-1α expression cassettes, respectively. Importantly, despite the induction of cytochrome *c* expression by PGC-1α that has been reported in other experimental contexts (Fig. 5B), no change in cytochrome *c* release was observed in ARPE-19 cells with ectopic elevation of PGC-1α (Fig. 5C). As expected, treatment of the ARPE-19 cells with actinomycin D or camptothecin caused elevated cytochrome *c* release and thus apoptosis in GFP-expressing control cells. However, when the cells were additionally expressing PGC-1α, this resulted in a significant reduction in cytochrome *c* release that was statistically undistinguishable from cytochrome *c* release in vehicle- treated control cells. (Fig. 5B).

### PGC-1α and PGC-1β are reduced in rd10 and VPP models of degenerative retinal disease

**Figure 6:**
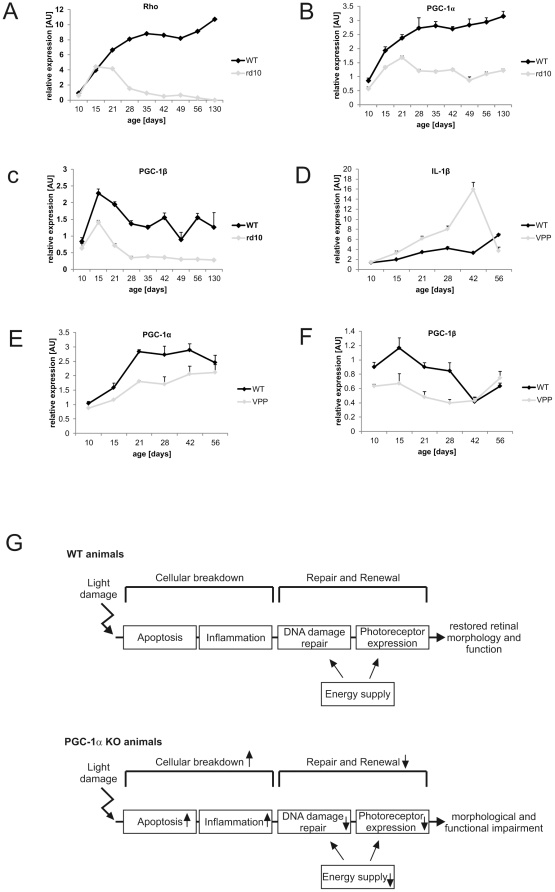
PGC-1α and PGC-1β are reduced in rd10 and VPP retinal degeneration models. Retina from rd10 and VPP mice was isolated at different PNDs (postnatal days) and checked for expression of genes via semi- quantitative real time PCR. β-actin was used as HKG. +SEM of n = 3 per time point. **A–C** rd10 mouse model: Rhodopsin (*Rho*) was used as control gene diminished in this condition. **D–F** VPP mouse model: Interleukin 1-β (*Il-1β*) was used as control gene marking the duration of the inflammatory response phase. **G** Loss of the PGC-1α gene in mice affects all the steps of the major cascade of events in light-induced retinal damage, repair and regeneration and consequently results in abnormal retinal morphology and function.

A wide array of retinal disease conditions is associated with increased apoptosis of photoreceptors, including *retinitis pigmentosa,* a multi- genetic hereditary retinal disease that is caused by mutations in genes implicated in photoreceptor and RPE function. *Retinitis pigmentosa* (RP) causes progressive vision loss by the degeneration of rods and cones [23]. Animal models to study RP include the rd10, the VPP and the *Nrl* KO mouse [18], [24]–[25]. Since apoptotic and other pathways involved in retinal damage in *retinitis pigmentosa* were affected by the PGC-1α gene knockout in light- exposed mice, we investigated whether the expression levels of PGC-1α were altered in these mouse models with age-dependent disease progression of retinal degeneration. In the WT animals, PGC-1α augmented with increasing age similar to *Rho (Rhodopsin)*, a marker of rod differentiation. In contrast, after an initial rise, PGC-1α expression reverted back to initial levels in the rd10 mouse (Fig. 6A, 6B). A similar decrease was observed for PGC-1β expression in the rd10 animals, where PGC-1β levels remained consistently lower compared to those of WT animals, even after the initial spike of PGC-1β expression around the age of 15 days (Fig. 6C). Any remaining amount of both coactivators was probably due to their presence in the GCL and INL which remained intact in all of the used models (Fig. 1B).

The VPP mouse suffers from a degeneration of rods and cones caused by three rhodopsin mutations (V20G, P23H, and P27L [VPP]) [24]. Both the rd10 and the VPP mouse models thus recapitulate the damage in rods and cones that is observed in human *retinitis pigmentosa*. Interleukin-1β (*Il-1β*), served as a control gene that is upregulated in the VPP mouse due to the inflammation in this model [26] (Fig. 6D), similar to what was shown for the inflammatory marker monocyte chemotactic protein 1(*Mcp-1*) [27]. Both PGC-1α and PGC-1β were expressed at a lower level in the VPP mouse compared to WT animals. Strikingly, convergence of both PGC-1α and PGC-1β expression in VPP and WT animals coincided with the relief of the inflammatory response in the VPP model implied by the strong decrease in *Il-1β* expression between day 42 and day 52. Thus, the apoptotic events seen in the VPP model that precede the inflammation directly correlated with reduced levels of PGC-1α and PGC-1β (Fig. 6D, 6E, 6F). Interestingly, PGC-1β was likewise reduced in the light- exposed PGC-1α knockout mice, which also exhibited retinal damage and dysfunction, as shown in the present manuscript (Fig. S1E).

## Discussion

The PGC-1 coactivators are key regulators of mitochondrial biogenesis and oxidative metabolism. Hence, our present observation of high expression of both PGC-1α and PGC-1β in the retina, a tissue with one of the highest energy demands, is not surprising. Moreover, the intraretinal expression pattern showing high levels of both coactivators in rod and cone photoreceptors reflects the high energetic requirement of phototransduction that to a large extent is met by oxidative phosphorylation [7]. Interestingly however, dark-adapted mice with an ablation of the PGC-1α gene in the retina display normal retinal morphology and function, even despite a notable reduction in metabolic gene expression in this experimental context. Lack of a prominent phenotype in PGC-1α loss-of-function settings in basal, unstressed conditions has also been observed in other organs in tissue-specific knockout mice [28], [13], [29] and in global knockout animals [19], [30]. Importantly, a more striking phenotypic dysregulation in these animal models is often induced by external stimuli such as fasting, cold exposure or exercise. Similarly, the absence of a functional PGC-1α gene has much more dramatic consequences in light-exposed compared to dark-adapted animals. Light stress and the resulting cellular damage in the retina results in a cascade of events aimed at destruction of damaged cells through apoptosis, removal of cellular debris, repair of DNA damage and restoration of phototransduction through increased expression of cone- and rod-specific genes (Fig. 6G). These processes require a considerable amount of energy, witnessed in the increased expression of genes implicated in oxidative phosphorylation in the WT light versus WT dark up (Table S1B). For example, components of the NADH dehydrogenase (*Ndufa*) and the *cytochrome c* oxidase (*Cox*) complexes are upregulated in this context. The PGC-1α KO animals, however, likely fail to meet the increased energy need (Table S1: KO li vs WT li down) and thus restoration of tissue homeostasis and function is hampered (Fig. 6G). In addition, apoptosis and the inflammatory response are enhanced in light-damaged retinae of PGC-1α knockout animals, while DNA damage repair and photoreceptor recovery are impaired in these mice (Table S2). Interestingly, a recent study analogously implied that PGC-1α dysregulation may be implicated in irradiation-caused DNA damage in the liver [31]. As a consequence, light-exposed retinae of PGC-1α knockout animals show morphological aberrations, specifically a destruction of the RIS and ROS. Interestingly, the thinning of the ONL in the KO animals seems to be primarily caused by a reduction in cell size and/or spacial cellular arrangement since the number of nuclei in the thinner ONL in the KO is not reduced, at least not at the time of the morphological analysis. This interesting observation will be the focus of future studies. As a result of the impairment in morphology and gene expression, light perception is significantly impaired in these mice, as suggested in particular by the reduced the rod-dependent, scotopic b-wave amplitude. In contrast, photopic light perception is unaltered: this difference between the drastic morphological phenotype and the apparently milder functional outcome could be due to the experimental setup. First, since mice were able to freely move during the light exposure, damage was not homogenous throughout the whole retina. Second, morphological and functional analysis were not performed at the same time after light exposure and were assessed in separate animals. Nevertheless, taken together, our data indicate an important role for PGC-1α in the protection against light-induced retinal damage, in providing energy for light perception and in repair and renewal of the photoreceptors of the retina.

The interpretation of our data obtained in global PGC-1α knockout animals could potentially be hampered by several drawbacks of this model. First, total ablation of the PGC-1α gene in many cases results in different, sometimes diametrically opposed phenotypic changes compared to those in tissue-specific knockouts [32]. For example, hepatic gluconeogenesis is constitutively elevated in one global PGC-1α knockout mouse model [19], whereas the fasting-dependent induction of gluconeogenesis is significantly blunted in liver-specific knockouts [29]. Similarly, fiber-type distribution in global knockout is unchanged compared to control mice, while AMP-dependent protein kinase (AMPK) is activated in this context [19], [33]. In contrast, a fiber-type switch towards glycolytic muscle fibers has been observed in muscle-specific PGC-1α knockouts that also exhibit normal AMPK activation [28], [13]. In both cases, the tissue-specific knockout models exhibit the phenotype that is expected based on *in vivo* gain-of-function experiments [34], [35]. Second, in a global PGC-1α knockout mouse, PGC-1α is obviously not only absent in retinal cells, but also other structures involved in light perception and vision. For example, PGC-1α knockout animals suffer from marked neurodegenerative events in the central nervous system [19]. Neuronal deficits, in particular in the visual cortex where PGC-1α expression has been reported [36], but also the optic nerve, would obviously also affect vision in these mice. Finally, it is difficult to interpret etiology and causality underlying the pathological phenotype in the global PGC-1α knockout animals with a life-long absence of a functional PGC-1α gene. To circumvent all of these potentially confounding problems of global, non-inducible PGC-1α gene ablation and to test whether apoptosis is directly affected by PGC-1α and not altered as a consequence of an energy crisis, we tested whether acute modulation of PGC-1α levels in an isolated system of RPE cells recapitulates our observations made in light-exposed PGC-1α knockout mice exhibiting increased levels of apoptosis *in vivo*. Our experiments in the ARPE-19 cell line clearly show a strong anti-apoptotic effect of ectopically expressed PGC-1α against two different pharmacological triggers of apoptosis, actinomycin D and camptothecin. PGC-1α expression has been associated with decreased apoptosis in different cellular contexts, including skeletal muscle [37], aortic endothelial cells [38] and decreased retinal capillary cell apoptosis after statin treatment [10]. In contrast, PGC-1α regulates intestinal epithelial cell fate in part by promoting apoptosis in the intestinal epithelial surface [39]. We now show for the first time that loss-of-function of PGC-1α results in increased apoptosis in the retina *in vivo* and that increased levels of PGC-1α exert an anti-apoptotic effect in retinal pigment epithelial cells.

Our tissue culture data thus not only confirm the direct involvement of loss-of-function of PGC-1α on apoptosis in the ONL *in vivo*, but together with the observation of reduced PGC-1α and PGC-1β expression in mouse models for *retinitis pigmentosa* also indicate that elevation of PGC-1α might be a valid approach to prevent retinal damage and also promote recovery in retinal diseases through increase in ATP generation. Future studies should thus aim at elucidating the therapeutic efficacy of normalization of PGC-1α levels with viral vectors or pharmacological tools on retinal degeneration in *retinitis pigmentosa* and other retinal pathologies.

## Methods

### Animals

All of the animal experiments have been approved by the institution and authorities of the cantons Basel-Stadt and Zurich in Switzerland as well as the institutional and federal authorities in Germany (no. 114/2010) and performed according to the statement of “The Association for Research in Vision and Ophthalmology” for the use of animals in research. Wildtype C57 BL/6 NRj mice were purchased from Janvier (Le Genest St. Isle, France). C57 BL/6 PGC-1α global KO mice were a kind gift of Bruce M. Spiegelman, Dana- Farber Cancer Institute and Department of Cell Biology, Harvard Medical School (Boston, MA). Rd10 ( = retinal degeneration 10) mice were purchased from Jackson Lab (Bar Harbor, Maine). VPP ( = mutant opsin transgene) mice were obtained from Muna Naash, University of Oklahoma (Oklahoma City, Oklahoma). *Nrl* (Neural retinal leucine zipper) KO mice were purchased from the University of Michigan (Ann Arbor, Michigan). Mice were maintained in a 12 h∶12 h light-dark cycle (60 lux).

### Light exposure

Six to thirteen- week old male C57 BL/6 PGC-1α global KO mice and wildtype C57 BL/6 NRj mice were dark adapted overnight (16 hrs). Pupils were dilated with 1% cyclogyl (Alcon, Cham, Switzerland) and 5% phenylephrine (Ciba Vision, Niederwangen, Switzerland) for 45 min. before light exposure. Mice were exposed to 15'000 lux of light for two hours. Following overnight recovery in darkness, mice were analyzed at time points indicated in the results section. Mice that were dark adapted and not exposed to light served as controls.

### Morphology and cell death detection ELISA

Following a 24 hours recovery period after light exposure, eyes were fixed in 2.5% glutaraldehyde in 0.1 M cacodylate buffer (pH 7.3) at 4°C overnight as a preparation for morphological analysis. The superior and inferior retinae were prepared, washed in cacodylate buffer, incubated in osmium tetroxide for 1 hour, dehydrated graded in alcohol series. Epon 812 sections (0.5 µM) from the lower central retina were counter-stained with methylene blue.

Cell death was quantified 24 hours after light exposure in isolated retinas using the ELISA-based cell death detection kit (Roche Diagnostics, Mannheim, Germany). Free nucleosomes, a hallmark of apoptosis in the retina and other tissues, were quantified using anti-histone antibodies in the KO and WT retinae. This method allows quantification of internucleosomal degradation of genomic DNA during apoptosis in proliferating and non-proliferating cells while excluding necrotic DNA.

### Laser capture microdissection

Mice were sacrificed, eyes enucleated, embedded in tissue freezing medium (Leica Microsystems Nussloch, Germany) and frozen in 2-methylbutane bath cooled by liquid nitrogen. Retinal sections (20 µM) were fixed (5 min. acetone), air-dried (5 min.) and dehydrated (30 sec 100% ethanol, 5 min. xylene). Microdissection was performed using an Arcturus XT Lasercapture device (Molecular devices, Silicon Valley, CA).

### Gene Expression Studies

RNA was isolated by pooling 2 retinas from 1 animal using the miRNeasy Mini Kit from Qiagen. 1 µg of RNA was employed for cDNA synthesis using Super Script Reverse Transcriptase II (Invitrogen) and random hexamer primers (Promega, Madison, USA). Real-time PCR analysis (Power SYBR Green Master Mix, Applied Biosystems, Carlsbad, California, USA) was performed either by using the LightCycler 480 Sybr Green I Master Kit and a LightCycler 480 instrument (Roche, Mannheim, Germany) or by Power SYBR Green Master Mix using the StepOnePlus sequence detector (Applied Biosystems, Carlsbad, California, USA). Relative expression levels were calculated with the ΔΔCt method and normalized to the expression of β-actin or 18S ribosomal RNA (18SrRNA) with primers indicated in Table S3.

### Cell culture and adenoviral infection

ARPE-19 cells were seeded at 2×10E5 cells per well of a 12 well plate in 50% F-12/50% DMEM medium supplemented with 1% Penicillin/Streptomycin. 24 h afterward, cells were infected with adenoviral constructs encoding GFP and PGC-1α, respectively.

### Apoptosis induction and cell death detection

48 h after adenoviral infection, cells were treated with Z-VAD-FMK (Peptanova, Sandhausen, Germany) for 60 min to prevent cell detachment. Apoptosis inducers Camptothecin or Actinomycin D (final concentration: 20 µM) were added. 24 h later, cells were fixed with 4% paraformaldehyde in PBS, washed with PBS and stained with anti-cytochrome *c* (BD biosciences). Released cytochrome *c* was quantified using fluorescence microscopy.

### ERG measurements

Electroretinograms (ERGs) were recorded according to previously described procedures [40]. Mice that were light exposed as described above were analyzed 10 days after light damage. Non-exposed mice served as controls. All mice were dark- adapted overnight prior to ERG analysis. Mice were then anaesthetized with ketamine (66.7 mg/kg body weight) and xylazine (11.7 mg/kg body weight). Pupils were dilated and single flash ERG responses were obtained under dark-adapted (scotopic) and light-adapted (photopic) conditions. Light adaptation was accomplished with a background illumination of 30 candela (cd) per square meter starting 10 min before recording. Single white-flash stimulus intensity ranged from −4 to 1.5 log cd·s/m2 under scotopic and from −2 to 1.5 log cd·s/m2 under photopic conditions.

### Spectral domain optical coherence tomography (SD-OCT)

Eyes were subjected to SD-OCT using the commercially available Spectralis™ HRA+OCT device from Heidelberg Engineering featuring a broadband superluminescent diode at λ = 870 nm as low coherent light source [41].

### Gene expression profiling

RNA was isolated as described above (Gene expression studies) and 4.25 ug of cDNA from the mouse retinae was hybridized to the Affymetrix Gene Chip Mouse 1.0 ST at the in-house microarray facility service. Microarray CEL output files were analyzed at the Functional Genomics Center Zurich, Winterthurerstrasse 190, CH-8057 Zurich. Statistical significance was calculated using the ANOVA test, Benjamin Hochberg corrected with the PARTEK and Bioconductor software suites. Thresholds for changes in gene expression set at 1.2 (0.2× upregulated) or 0.87 (0.2× downregulated). p≤0.05 = *, p≤0.01 = **, p≤0.001 = ***.

## Supporting Information

Figure S1 **Gene expression analysis of WT and PGC-1α KO mice in light and dark.** (**a**) Microarray analysis of light and dark exposed PGC-1α KO and C57BL/6j WT control mice; Gene expression changes in the four comparisons: 18sRNA was used as HKG. +SEM for n = 3; * = p<0.05; ** = p<0.01; *** = p<0.001. Statistical significance was calculated using two tailed Student's T test (**b**) phototransduction genes, (**c**) pro and anti-apoptotic genes, (**d**) inflammatory genes and (**e**) PGC-1α and PGC-1β gene expression.
(PDF)

Table S1 **Microarray analysis of light and dark exposed PGC-1α KO and C57BL/6j WT control mice: top genes and pathways.** (**a**) Top 10 genes most highly up-/downregulated in their mRNA expression of PGC-1α KO and C57BL/6j WT mice, dark vs light exposed. (**b**) Top 10 pathways up-/downregulated in their mRNA expression of PGC-1α KO and C57BL/6j WT mice, dark vs light exposed. n = 3; * = p<0.05; ** = p<0.01; *** = p<0.001. Statistical significance was calculated using ANOVA test, Benjamin Hochberg corrected. Thresholds for changes in gene expression set at 1.2 (0.2× upregulated) or 0.87 (0.2× downregulated).
(PDF)

Table S2 **Microarray analysis of light and dark exposed PGC-1α KO and C57BL/6j WT control mice.** Significant changes in genes involved in apoptosis, DNA repair, inflammation and phototransduction. green = upregulated; red = downregulated (**a**) pro and anti-apoptotic, DNA repair genes (**b**) phototransduction and protein folding genes (**c**) ECM breakdown and inflammatory genes.
(PDF)

Table S3 **List of primers for semi-quantitative real time PCR analysis.**
(PDF)
