# Supplementary material for: PGC-1α Determines Light Damage Susceptibility of the Murine Retina
Source: PLoS One. 2012 Feb 13;7(2):e31272. doi: 10.1371/journal.pone.0031272 (PMC3278422; doi:10.1371/journal.pone.0031272)
Supplement: Figure S1 — Gene expression analysis of WT and PGC-1α KO mice in light and dark. (a) Microarray analysis of light and dark exposed PGC-1α KO and C57BL/6j WT control mice; Gene expression changes in the four comparisons: 18sRNA was used as HKG. +SEM for n = 3; * = p<0.05; ** = p<0.01; *** = p<0.001. Statistical significance was calculated using two tailed Student's T test (b) phototransduction genes, (c) pro and anti-apoptotic genes, (d) inflammatory genes and (e) PGC-1α and PGC-1β gene expression. (PDF) [file pone.0031272.s001.pdf]

Fig. S1

A Phototransduction

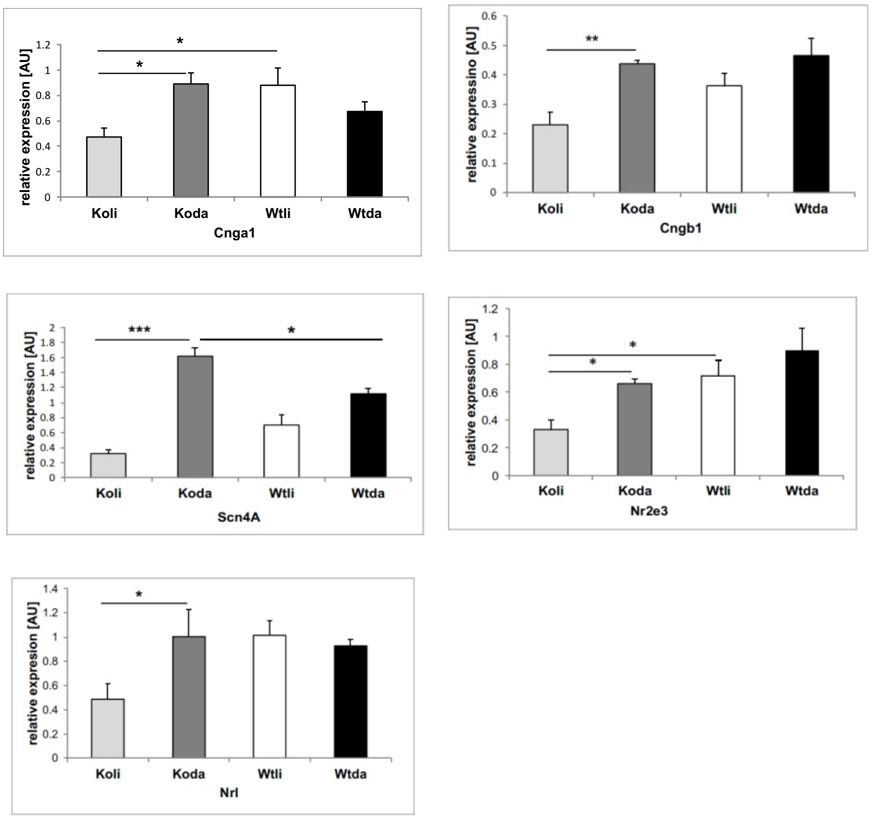

B Pro apoptotic

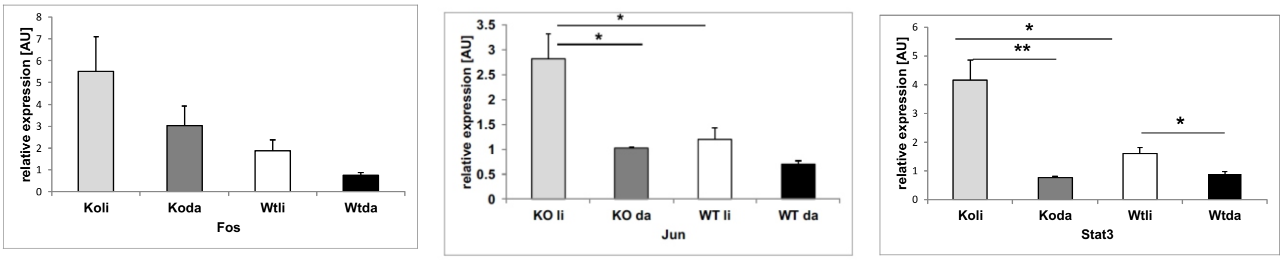

C Anti apoptotic

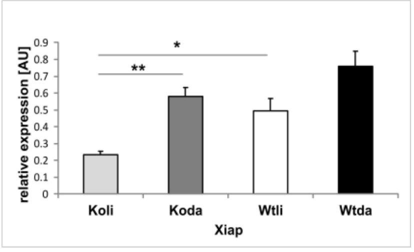

D PGC-1α and PGC-1β

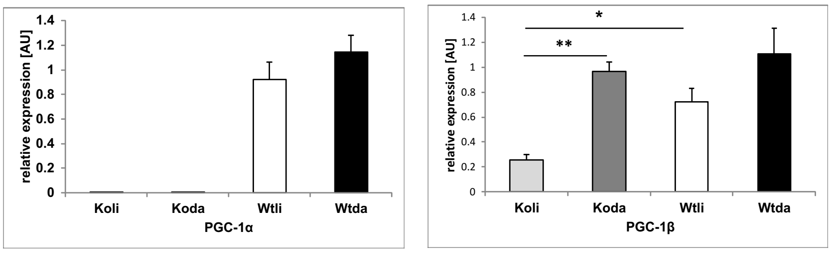

E

number of genes changed: light vs dark		
	KO	WT
↑	1410	1002
↓	1504	761

number of genes changed: KO vs WT		
	light	dark
↑	897	167
↓	877	335
