# Supplementary material for: PGC-1α Determines Light Damage Susceptibility of the Murine Retina
Source: PLoS One. 2012 Feb 13;7(2):e31272. doi: 10.1371/journal.pone.0031272 (PMC3278422; doi:10.1371/journal.pone.0031272)
Supplement: Table S1 — Microarray analysis of light and dark exposed PGC-1α KO and C57BL/6j WT control mice: top genes and pathways. (a) Top 10 genes most highly up-/downregulated in their mRNA expression of PGC-1α KO and C57BL/6j WT mice, dark vs light exposed. (b) Top 10 pathways up-/downregulated in their mRNA expression of PGC-1α KO and C57BL/6j WT mice, dark vs light exposed. n = 3; * = p<0.05; ** = p<0.01; *** = p<0.001. Statistical significance was calculated using ANOVA test, Benjamin Hochberg corrected. Thresholds for changes in gene expression set at 1.2 (0.2× upregulated) or 0.87 (0.2× downregulated). (PDF) [file pone.0031272.s002.pdf]

Table S1

A

KO li vs KO da down			
gene ID	gene name	pval	ratio
ENSMUSG00000086503	AL669964.8	2.06E-08	0.005687
ENSMUSG00000006154	Eps8l1	0.000298	0.310375
ENSMUSG00000031966	Glb1l3	0.042325	0.345601
ENSMUSG00000019232	Agxt2l1	0.01089	0.395672
ENSMUSG00000065619	mmu-mir-183	0.00074	0.411426
ENSMUSG00000022899	Slc15a2	0.042768	0.434437
ENSMUSG00000060890	Arr3	0.001724	0.447277
ENSMUSG00000063590	Gm5631	0.011673	0.453493
ENSMUSG00000001027	Scn4a	0.001642	0.453723
ENSMUSG00000074930	RP23-224B9.1	0.003755	0.454678

KO li vs KO da up			
gene ID	gene name	pval	ratio
ENSMUSG00000069049	Eif2s3y	2.35E-07	54.80594
ENSMUSG00000068457	Uty	2.81E-07	35.7843
ENSMUSG00000069045	Ddx3y	2.86E-06	13.24099
ENSMUSG00000056673	Kdm5d	2.16E-07	9.542695
ENSMUSG00000021091	Serpina3n	6.66E-06	7.656752
ENSMUSG00000015312	Gadd45b	0.001278	7.013121
ENSMUSG00000000982	Ccl3	0.002205	6.726247
ENSMUSG00000028270	Gbp2	2.86E-05	6.570339
ENSMUSG00000041782	Lad1	0.000118	5.721613
ENSMUSG00000030111	A2m	0.001193	5.592195

KO da vs WT da down			
gene ID	gene name	p val	ratio
ENSMUSG00000042210	Abhd14a	2.07807E-05	0.193843
ENSMUSG00000075751	SNORD115	0.00054873	0.249685
ENSMUSG00000048758	Rpl29	0.001339296	0.252956
ENSMUSG00000044573	Acp1	0.000374379	0.271788
ENSMUSG00000034248	Slc25a37	0.000491269	0.389097
ENSMUSG00000074407	AC124577.1	0.006878776	0.42765
ENSMUSG00000087178	AC102121.3	0.00136835	0.429943
ENSMUSG00000015656	Hspa8	0.000325941	0.459108
ENSMUSG00000067536	Dnahc7b	0.001128195	0.470358
ENSMUSG00000066705	Fxyd6	0.000461585	0.478773

KO da vs WT da up			
gene ID	gene name	p val	ratio
ENSMUSG00000060177	Klk1b22	0.019040589	14.68375
ENSMUSG00000035296	Sgcg	4.08404E-05	6.800434
ENSMUSG00000067299	Crygd	0.03520007	4.578564
ENSMUSG00000038418	Egr1	0.000184156	3.668763
ENSMUSG00000075830	SNORD115	0.000895339	3.569132
ENSMUSG00000075786	SNORD115	0.000175157	3.160565
ENSMUSG00000042073	Abhd14b	0.000261255	3.072122
ENSMUSG00000039217	Il18	0.001231077	2.637179
ENSMUSG00000056199	AC130827.2	0.002466451	2.45678
ENSMUSG00000052143	AC156940.1	1.64301E-05	2.258859

WT li vs WT da down			
gene ID	gene name	pval	ratio
ENSMUSG00000086503	AL669964.8	3.37E-07	0.005715
ENSMUSG00000048758	Rpl29	0.000464	0.167101
ENSMUSG00000042210	Abhd14a	6.49E-07	0.208578
ENSMUSG00000044573	Acp1	9.95E-05	0.218836
ENSMUSG00000006154	Eps8l1	0.00327	0.318603
ENSMUSG00000034248	Slc25a37	0.000923	0.413001
ENSMUSG00000076867	A630098A13Rik	0.005688	0.439523
ENSMUSG00000025794	Rpl14	0.020493	0.464948
ENSMUSG00000005994	Tyrp1	0.006139	0.487175
ENSMUSG00000022129	Dct	0.034269	0.490081

WT li vs WT da up			
gene ID	gene name	p val	ratio
ENSMUSG00000069049	Eif2s3y	1.00932E-07	60.1737237
ENSMUSG00000068457	Uty	1.6419E-07	37.7591677
ENSMUSG00000069045	Ddx3y	1.24415E-05	14.3821267
ENSMUSG00000056673	Kdm5d	3.44951E-06	8.32423296
ENSMUSG00000035296	Sgcg	3.30188E-05	7.00414513
ENSMUSG00000042073	Abhd14b	1.02711E-05	3.23125544
ENSMUSG00000020932	Gfap	0.01813095	2.4320601
ENSMUSG00000038418	Egr1	0.006573966	2.38133213
ENSMUSG00000034855	Cxcl10	0.000221406	2.3193745
ENSMUSG00000071984	Fndc1	0.008513625	2.19287216

KO li vs WT li down			
gene ID	gene name	pval	ratio
ENSMUSG00000031966	Glb1l3	0.011341	0.200791
ENSMUSG00000071984	Fndc1	0.017437	0.420968
ENSMUSG00000074407	AC124577.1	0.013583	0.42309
ENSMUSG00000072844	G530011O06Rik	0.005516	0.457092
ENSMUSG00000025450	AC163689.1	0.001251	0.477825
ENSMUSG00000015656	Hspa8	0.003025	0.480434
ENSMUSG00000035296	Sgcg	0.008462	0.483232
ENSMUSG00000005233	Spc25	0.034111	0.511731
ENSMUSG00000037112	Sik2	0.002234	0.514723
ENSMUSG00000027360	Hdc	0.002264	0.538682

KO li vs WT li up			
gene ID	gene name	p val	ratio
ENSMUSG00000060177	Klk1b22	0.01315403	14.15191
ENSMUSG00000029816	Gpnmb	0.00063578	5.6861176
ENSMUSG00000030111	A2m	0.00503549	4.1088189
ENSMUSG00000000982	Ccl3	0.01009673	3.8950643
ENSMUSG00000015312	Gadd45b	0.00889059	3.8511659
ENSMUSG00000026822	Lcn2	0.00337735	3.8338973
ENSMUSG00000021091	Serpina3n	0.0162945	3.6072455
ENSMUSG00000023078	Cxcl13	0.01890613	3.2662722
ENSMUSG00000041782	Lad1	0.00512352	3.0965626
ENSMUSG00000005087	Cd44	0.01043177	2.9783082

B

KO li vs KO da down	
pathway name	pValue
Neurophysiological process _Visual perception	2.59E-08
Protein folding_Membrane trafficking and signal transduction of G-alpha (i) heterotrimeric G-protein	5.13E-08
Cardiac Hypertrophy_NF-AT signaling in Cardiac Hypertrophy	5.92E-08
Neurophysiological process_ACM regulation of nerve impulse	4.98E-07
Development_Role of HDAC and calcium/calmodulin-dependent kinase (CaMK) in control of skeletal myogenesis	7.27E-07
Immune response_Function of MEF2 in T lymphocytes	1.54E-06
Cell adhesion_ECM remodeling	2.58E-06
Neurophysiological process_ACM1 and ACM2 in neuronal membrane polarization	3.9E-06
Muscle contraction_ GPCRs in the regulation of smooth muscle tone	1.29E-05
Signal transduction_cAMP signaling	1.45E-05

KO li vs KO da up	
pathway name	pValue
Apoptosis and survival_Endoplasmic reticulum stress response pathway	1.05E-14
Cytoskeleton remodeling_Cytoskeleton remodeling	5.92E-13
Immune response_Oncostatin M signaling via MAPK in human cells	9.01E-13
Immune response_Oncostatin M signaling via MAPK in mouse cells	2.39E-11
Cytoskeleton remodeling_TGF, WNT and cytoskeletal remodeling	6.61E-11
Cell adhesion_Chemokines and adhesion	1.43E-10
G-protein signaling_G-Protein alpha-12 signaling pathway	9.51E-10
Immune response_ETV3 affect on CSF1-promoted macrophage differentiation	1.39E-09
Apoptosis and survival_BAD phosphorylation	2.37E-09
G-protein signaling_Ras family GTPases in kinase cascades (scheme)	3.57E-09

KO dark vs WT dark down	
pathway name	pValue
Oxidative phosphorylation	5.02E-16
Ubiquinone metabolism	4.8E-09
TCA	2.06E-05
Transcription_Transcription regulation of aminoacid metabolism	0.000839
Transcription_Role of VDR in regulation of genes involved in osteoporosis	0.002058
Aminoacyl-tRNA biosynthesis in cytoplasm	0.002839
Aminoacyl-tRNA biosynthesis in cytoplasm/ Rodent version	0.003044
Leucune, isoleucine and valine metabolism	0.005915
Cytoskeleton remodeling_Neurofilaments	0.006587
Unsaturated fatty acid biosynthesis	0.011499

KO dark vs WT dark up	
pathway name	pValue
Cytoskeleton remodeling_TGF, WNT and cytoskeletal remodeling	3.09E-07
Transcription_Androgen Receptor nuclear signaling	9.85E-07
Immune response_MIF - the neuroendocrine-macrophage connector	1.25E-06
Development_GM-CSF signaling	2.11E-05
Development_WNT signaling pathway. Part 2	3.6E-05
Development_Ligand-independent activation of ESR1 and ESR2	4.54E-05
Cytoskeleton remodeling_Cytoskeleton remodeling	4.58E-05
PGE2 pathways in cancer	5.03E-05
Development_TGF-beta-dependent induction of EMT via SMADs	5.2E-05
Development_Endothelin-1/EDNRA transactivation of EGFR	6.58E-05

WT li vs WT da down	
pathway name	pValue
Neurophysiological process_ACM regulation of nerve impulse	1.63E-06
Immune response_Function of MEF2 in T lymphocytes	2.67E-05
Transcription_Transcription regulation of aminoacid metabolism	4.49E-05
Development_Notch Signaling Pathway	4.63E-05
Development_Role of HDAC and calcium/calmodulin-dependent kinase (CaMK) in control of skeletal myogenesis	5.38E-05
Cardiac Hypertrophy_NF-AT signaling in Cardiac Hypertrophy	0.00027
Transcription_Ligand-Dependent Transcription of Retinoid-Target genes	0.000301
Development_Hedgehog signaling	0.000473
Transcription_Sin3 and NuRD in transcription regulation	0.000746
Protein folding_Membrane trafficking and signal transduction of G-alpha (i) heterotrimeric G-protein	0.000794

WT li vs WT da up	
pathway name	pValue
Transcription_P53 signaling pathway	7.82E-08
Oxidative phosphorylation	3.73E-06
Development_PDGF signaling via STATs and NF-kB	1.55E-05
Development_Role of IL-8 in angiogenesis	2.31E-05
Immune response_Oncostatin M signaling via JAK-Stat in mouse cells	2.66E-05
Development_TGF-beta-dependent induction of EMT via RhoA, PI3K and ILK.	2.91E-05
Development_Angiopoietin - Tie2 signaling	4.05E-05
Immune response_Oncostatin M signaling via MAPK in mouse cells	4.05E-05
Development_Slit-Robo signaling	5.15E-05
Cytoskeleton remodeling_Neurofilaments	6.17E-05

KO li vs WT li down	
pathway name	pValue
Neurophysiological process _Visual perception	1.48E-09
Oxidative phosphorylation	1.21E-05
Cell cycle_The metaphase checkpoint	0.000282
Cell cycle_Chromosome condensation in prometaphase	0.000389
DNA damage_ATM / ATR regulation of G2 / M checkpoint	0.00134
Cardiac Hypertrophy_Ca(2+)-dependent NF-AT signaling in Cardiac Hypertrophy	0.001668
Protein folding_Membrane trafficking and signal transduction of G-alpha (i) heterotrimeric G-protein	0.001822
Apoptosis and survival_Granzyme A signaling	0.002926
Atherosclerosis_Role of ZNF202 in regulation of expression of genes involved in Atherosclerosis	0.00294
Ubiquinone metabolism	0.003117

KO li vs WT li up	
pathway name	pValue
Cell adhesion_Chemokines and adhesion	7.3E-16
Cytoskeleton remodeling_TGF, WNT and cytoskeletal remodeling	1.23E-12
Cytoskeleton remodeling_Cytoskeleton remodeling	1.94E-12
Reproduction_GnRH signaling	3.91E-10
Neurophysiological process_Receptor-mediated axon growth repulsion	5.66E-10
Signal transduction_Calcium signaling	5.66E-10
Development_Regulation of epithelial-to-mesenchymal transition (EMT)	4.52E-09
Cell adhesion_Role of tetraspanins in the integrin-mediated cell adhesion	6.05E-09
Development_Slit-Robo signaling	1.13E-08
Cytoskeleton remodeling_Regulation of actin cytoskeleton by Rho GTPases	1.57E-08
