# Supplementary material for: PGC-1α Determines Light Damage Susceptibility of the Murine Retina
Source: PLoS One. 2012 Feb 13;7(2):e31272. doi: 10.1371/journal.pone.0031272 (PMC3278422; doi:10.1371/journal.pone.0031272)
Supplement: Table S2 — Microarray analysis of light and dark exposed PGC-1α KO and C57BL/6j WT control mice. Significant changes in genes involved in apoptosis, DNA repair, inflammation and phototransduction. green = upregulated; red = downregulated (a) pro and anti-apoptotic, DNA repair genes (b) phototransduction and protein folding genes (c) ECM breakdown and inflammatory genes. (PDF) [file pone.0031272.s003.pdf]

# Table S2

A

| *Koda vs Wtda* | *Koli vs Wtli* | *Koli vs KO da* | *Wtli vs Wtda* | *role* |
|---|---|---|---|---|
| *comparison1* | *comparison2* | *comparison3* | *comparison4* | |
| | | | | |
| | BCL-2 | BLC-2 | | anti apoptotic |
| | XIAP | | XIAP | anti apoptotic |
| | | | BAX | anti apoptotic |
| | | HRK | | anti apoptotic |
| | | RPS6KB1 | | anti apoptotic |
| | | MDM2 | MDM2 | anti apoptotic |
| | TIMP1 | TIMP1 | | anti apoptotic |
| PARK7 | | PARK7 | PARK7 | anti apoptotic |
| FASTK | | | FASTK | pro apoptotic |
| | BID | BID | | pro apoptotic |
| | | JAK2 | JAK2 | pro apoptotic |
| | NR4A1 | NR4A1 | NR4A1 | pro apoptotic |
| | CRADD | CRADD | | pro apoptotic |
| | TNFSF15 | TNFSF15 | TNFSF15 | pro apoptotic |
| | | | TRAF2 | pro apoptotic |
| | CASP7 | CASP7 | | pro apoptotic |
| | GZMA | GZMA | | pro apoptotic |
| ANP32A | ANP32A | | | pro apoptotic |
| | APAF-1 | APAF-1 | APAF-1 | pro apoptotic |
| | DAXX | DAXX | DAXX | pro apoptotic |
| | DOCK1 | DOCK1 | | pro apoptotic |
| | FAS | FAS | | pro apoptotic |
| | GADD45b | GADD45b | GADD45b | pro apoptotic |
| | p53 | p53 | | pro apoptotic |
| | TNFRSF1a | TNFRSF1a | | pro apoptotic |
| | | TRADD | | pro apoptotic |
| | CASPASE8 | CASPASE8 | | pro apoptotic |
| | | FOS | FOS | pro apoptotic |
| | | ERN1 | ERN1 | pro apoptotic |
| | | MAPK8ip2 | MAPK8ip2 | pro apoptotic |
| | | | BMF | pro apoptotic |
| | STAT1 | STAT1 | STAT1 | pro apoptotic |
| | JUN | JUN | | pro apoptotic |
| | STAT3 | STAT3 | STAT3 | pro apoptotic |
| | | | STAT4 | pro apoptotic |
| | STAT5 | STAT5 | STAT5 | pro apoptotic |
| | | | LIF | pro apoptotic |
| | Pxn | | Pxn | cell adhesion |
| POLD4 | | | POLD4 | D.N.A. repair |
| RECQL | | RECQL | | D.N.A. repair |
| TREX2 | | TREX2 | TREX2 | D.N.A. repair |
| | BLM | BLM | | D.N.A. repair |
| | FANCD2 | | | D.N.A. repair |
| | FANCE | | FANCE | D.N.A. repair |
| | FANCG | | FANCG | D.N.A. repair |
| | FANCL | | FANCL | D.N.A. repair |
| | MGMT | MGMT | | D.N.A. repair |
| | MMS21 (NSMCE2) | | MMS21 (NSMCE2) | D.N.A. repair |
| | RPA3 | | | D.N.A. repair |
| | | SMC5 | | D.N.A. repair |
| | | APEX (REF1) | | D.N.A. repair |
| | | ATM | ATM | D.N.A. repair |
| | BAP1 | BAP1 | BAP1 | D.N.A. repair |
| | | | XPC | D.N.A. repair |
| | | | POLD3 | D.N.A.repair |
| | | POLD1 | | D.N.A.repair |
| | RECQL5 | | | D.N.A.repair |

Table S2

B

| *Koda vs Wtda* | *Koli vs Wtli* | *Koli vs KO da* | *Wtli vs Wtda* | *role* |
|---|---|---|---|---|
| *comparison1* | *comparison2* | *comparison3* | *comparison4* | |
| | HSP90ab1 | HSP90ab1 | HSP90ab1 | protein folding |
| | | DERL-1 | | protein folding |
| | | eIF2AK3 | eIF2AK3 | protein folding |
| | | | CEBPZ | protein folding |
| | | | UBC7 | protein folding |
| | | UBE3C | UBE3C | protein folding |
| | LMNB1 | | LMNB1 | structure; scaffold |
| AIPL1 | | AIPL1 | | phototransduction |
| | ARR3 | ARR3 | ARR3 | phototransduction |
| CABP1 | CABP1 | CABP1 | CABP1 | phototransduction |
| | CNGA1 | CNGA1 | | phototransduction |
| CNGB1 | CNGB1 | CNGB1 | | phototransduction |
| | GJC2 | | | phototransduction |
| | | CRX | | phototransduction |
| | GRK1 | | GRK1 | phototransduction |
| | GUCY2D | | GUCY2D | phototransduction |
| | GUCY2F | | GUCY2F | phototransduction |
| | NR2E3 | | | phototransduction |
| | NRL | NRL | | phototransduction |
| | OPN1MW | OPN1MW | OPN1MW | phototransduction |
| | PDE6A | | PDE6A | phototransduction |
| | PDE6B | PDE6B | | phototransduction |
| | | PDE6D | | phototransduction |
| | PDE6G | PDE6G | | phototransduction |
| PDE6H | PDE6H | PDE6H | PDE6H | phototransduction |
| | RBP3 | RBP3 | | phototransduction |
| | RGS9BP | | RGS9BP | phototransduction |
| RDH14 | | | | phototransduction |
| | RCVRN | RCVRN | | phototransduction |
| | GUCA1A | GUCA1A | GUCA1A | phototransduction |
| | RGS9 | RGS9 | | phototransduction |
| | RHOD | RHOD | | phototransduction |
| | | | RLBP1 | phototransduction |
| RPGRIP1 | RPGRIP1 | RPGRIP1 | RPGRIP1 | phototransduction |
| | SLC24A1 | SLC24A1 | SLC24A1 | phototransduction |
| | | | Slc24A2 | phototransduction |

# Table S2

C

| *Koda vs Wtda* | *Koli vs Wtli* | *Koli vs KO da* | *Wtli vs Wtda* | *role* |
|---|---|---|---|---|
| *comparison1* | *comparison2* | *comparison3* | *comparison4* | |
| | | | | |
| | MMP14 | MMP14 | | ECM breakdown |
| | | MMP9 | | ECM breakdown |
| | | MIF | | inflammatory |
| | | PLG (Angiostatin) | | inflammatory |
| | CDC25C | | CDC25C | inflammatory |
| | | CREB3 | CREB3 | inflammatory |
| | MAP3K1 | MAP3K1 | MAP3K1 | inflammatory |
| | IL-1a | IL-1a | | inflammatory |
| | | | IRAK1 | inflammatory |
| | IRAK2 | | IRAK2 | inflammatory |
| | ITGBI | ITGBI | | inflammatory |
| | VEGF-A | | | inflammatory |
| | A2M | A2M | | inflammatory |
| | | | CCL2 | inflammatory |
| | | CREB3 | CREB3 | inflammatory |
| | | MYD88 | | inflammatory |
| | | NFkb | NFkb | inflammatory |
| | OSMR | OSMR | OSMR | inflammatory |
| | MAPK14 | MAPK14 | | inflammatory |
| | DUSP16 | DUSP16 | DUSP16 | inflammatory |
| | | MTA2 | MTA2 | inflammatory |
| | OSM | | OSM | inflammatory |
| | RelA | RelA | RelA | inflammatory |
| | SENP2 | | SENP2 | inflammatory |
| | SMAD3 | SMAD3 | SMAD3 | inflammatory |
| | | | STAT4 | inflammatory |
| | XBPI | XBPI | XBPI | inflammatory |
| | | | TLR2 | inflammatory |
| | VCAM1 | VCAM1 | VCAM1 | inflammatory |
| | OSMR | OSMR | OSMR | inflammatory |
| MFN2 | MFN2 | MFN2 | MFN2 | mitochondria |
