# Supplementary material for: PGC-1α Determines Light Damage Susceptibility of the Murine Retina
Source: PLoS One. 2012 Feb 13;7(2):e31272. doi: 10.1371/journal.pone.0031272 (PMC3278422; doi:10.1371/journal.pone.0031272)
Supplement: Table S3 — List of primers for semi-quantitative real time PCR analysis. (PDF) [file pone.0031272.s004.pdf]

Table S3

Primer name	Sequence 5’ => 3’
PGC-1α_ex3-5-F	AGCCGTGACCACTGACAACGAG
PGC-1α_ex3-5-R	GCTGCATGGTTCTGAGTGCT AAG
PGC-1β-F	GGCAGGTTCAACCCCGA
PGC-1β-R	CTTGCTAACATCACAGAGGA TATCTTG
Gnat1-F	GAGGATGCTGAGAAGGATGC
Gnat1-R	TGAATGTTGAGCGTGGTCAT
β- Actin-F	CAACGGCTCCGGCATGTGC
β-Actin-R	CTCTTGCTCTGGGCCTCG
18S rRNA-F	AGTCCCTGCCCTTTGTACAC A
18S rRNA-R	CGATCCGAGGGCCTCACTA
Bcl2-F	ACCGGGAGATCGTGATGAAG
Bcl2-R	ATCTCCAGCATCCCACTCGT A
Fas-F	GCTGGCTCACAGTTAAGAGTTC ATAC
Fas-R	ACCCGCCTCCTCAGCTTT
Stat3-F	TGCGGAGAAGCATTGTGAGT G
Stat3-R	TTTTCCAGACGTGTCCAGGC AGATG
Opn1mw-F	TGTACATGGTCAACAATCGG A
Opn1mw-R	ACACCATCTCCAGAATGCAA G
Cycs-F	GGCGTGTCCTTGGACTTAGA
Cycs-R	TGCCTTTCTCAACATCACCC
Cox5b-F	GCTCCATGGCATCTGGA
Cox5b-R	ATGGGTCCAGTCCCTTCTTT
Rho-F	CTTCACCTGGATCATGGCGT T
Rho-R	TTCGTTGTTGACCTCAGGCTTG
Mcp-1-F	GGCTCAGCCAGATGCAGTTA
Mcp-1-R	CTGCTGCTGGTGATCCTCTT
Il-1β-F	GCAGGCAGTATCACTCATTG
